# Comparison of Analysis Tools for miRNA High Throughput Sequencing Using Nerve Crush as a Model

**DOI:** 10.3389/fgene.2013.00020

**Published:** 2013-03-01

**Authors:** Raghu Prasad Rao Metpally, Sara Nasser, Ivana Malenica, Amanda Courtright, Elizabeth Carlson, Layla Ghaffari, Stephen Villa, Waibhav Tembe, Kendall Van Keuren-Jensen

**Affiliations:** ^1^Collaborative Bioinformatics Center, Translational Genomics Research InstitutePhoenix, AZ, USA; ^2^Neurogenomics, Translational Genomics Research InstitutePhoenix, AZ, USA; ^3^Medical School, University of California San FranciscoSan Francisco, CA, USA

**Keywords:** miRNA, small RNA, nerve injury, analysis, next generation sequencing, plasma, muscle

## Abstract

Recent advances in sample preparation and analysis for next generation sequencing have made it possible to profile and discover new miRNAs in a high throughput manner. In the case of neurological disease and injury, these types of experiments have been more limited. Possibly because tissues such as the brain and spinal cord are inaccessible for direct sampling in living patients, and indirect sampling of blood and cerebrospinal fluid are affected by low amounts of RNA. We used a mouse model to examine changes in miRNA expression in response to acute nerve crush. We assayed miRNA from both muscle tissue and blood plasma. We examined how the depth of coverage (the number of mapped reads) changed the number of detectable miRNAs in each sample type. We also found that samples with very low starting amounts of RNA (mouse plasma) made high depth of mature miRNA coverage more difficult to obtain. Each tissue must be assessed independently for the depth of coverage required to adequately power detection of differential expression, weighed against the cost of sequencing that sample to the adequate depth. We explored the changes in total mapped reads and differential expression results generated by three different software packages: miRDeep2, miRNAKey, and miRExpress and two different analysis packages, DESeq and EdgeR. We also examine the accuracy of using miRDeep2 to predict novel miRNAs and subsequently detect them in the samples using qRT-PCR.

## Introduction

miRNAs are small non-coding RNAs ∼22 nucleotides in length that regulate gene expression by altering mRNA stability and transcription. miRNAs are thought to regulate at least 30% of genes and are involved in most cellular processes (Ebert and Sharp, 2012; Espinoza-Lewis and Wang, 2012; Ponomarev et al., 2012), including disease (Provost, 2010; Schroen and Heymans, 2012; Shantikumar et al., 2012). As a result, miRNA expression profiling studies have been effective in identifying specific miRNA signatures in a variety of developmental stages and diseases (Natarajan et al., 2012; Nikitina et al., 2012; Pritchard et al., 2012; Weiland et al., 2012). Most of these studies have occurred in the fields of cancer research, diabetes, and cardiovascular disease. Studies examining miRNA changes associated with neurological disease and injury have lagged behind.

The lag in miRNA studies of neurological and neurodegenerative disease is in part due to our inability to directly test affected tissues and cells from living subjects. Indirect sampling of miRNAs in blood and cerebrospinal fluid, because of their small amounts of total RNA, have not easily leant themselves for profiling by next generation sequencing (NGS). Recent advances in library sample preparation have introduced new and sensitive protocols that have improved our ability to differentiate changes in miRNA expression levels, even from samples with low amounts of RNA. Deep sequencing allows for massive parallel quantification and evaluation of the miRNA composition in a large number of samples at one time. Using the Illumina NGS platform, we routinely barcode and sequence up to 175 small RNA samples per flow cell on the HiSeq 2000. The bias associated with multiplexing samples has also made vast improvements in the new types of sample preparation. Instead of ligating a barcode directly to the miRNA and introducing bias through ligation efficiency, the same adaptor sequence is ligated to all of the samples and the individual barcode is introduced by PCR, resulting in virtually no bias (Post Amplification Ligation-Mediated multiplexing; Van Nieuwerburgh et al., 2011). The technique accommodates many different sample types (Osanto et al., 2012; Semenov et al., 2012; Wang et al., 2012), making NGS of miRNAs increasingly more accessible, cost efficient, and quantitative.

As the protocols for miRNA library preparation for deep sequencing have improved, the real challenge has become how to appropriately adapt, and integrate better analysis tools. As we began our experiments using small RNA NGS data, we were uncertain how many initial reads and mapped reads per sample library we should acquire, what variation in the number of initial vs. mapped reads to expect between biological replicates and from different sample types – for example, tissue vs. acellular fluid. We needed to determine how many miRNAs we could detect in tissue vs. plasma samples, which alignment and miRNA detection software to choose, and what analysis software to use. We describe our experiences using three different software programs (miRNA Key, miRDeep2, and miR Express) and two different analysis packages to detect significant, differentially expressed miRNAs using EdgeR and DESeq. We used experimental data from acute nerve injury. We sequenced miRNA from whole gastrocnemius muscle and blood plasma collected from mice 7 days after they received a sciatic nerve crush or an identical surgical procedure minus the crushed nerve (sham-surgery). In this report, we discuss our experience sequencing these two sample types and taking our data through all three alignment tools and both analysis programs, and finally we present the differentially expressed miRNAs identified by each pipeline. Although each lab, each tissue, and each experimental manipulation will have to be evaluated individually for its own inherent variability, there are common conclusions that can be drawn.

## Materials and Methods

### Animal surgery and handling

Experimental procedures and animal handling were performed using protocols approved by the Institutional Animal Care and Use Committee at the Barrow Neurological Institute, St. Joseph’s Hospital, and Medical Center.

Six-week-old C57BL6 mice were used for all experiments. Ten mice were deeply anesthetized using intraperitoneal injections of 80 mg/kg ketamine and 10 mg/kg xylazine, in addition to atropine (0.02 mg/kg) to reduce bronchial secretion. Animals were placed on their left side to expose the right Quadriceps muscle. The hair on the thigh was clipped and the area thoroughly sterilized. An incision through the skin was made using a scalpel. A blunt dissection was made into the right thigh, between the Gluteus Maximus and Quadriceps muscles (procedure according to Luís et al., 2007; Mazzer et al., 2008). A Schwartz micro clip (non-serrated) with ∼795 gm of occluding pressure (Roboz) was used to clamp the sciatic nerve for 10 s. This was enough time and pressure to see significant flattening of the sciatic nerve. The muscle and skin were then closed with 5/0 sutures. Animals were given 7.5 mg/kg Ibuprofen orally for 3 days following the procedure. Seven days post-procedure, the animals were anesthetized, and blood removed by cardiac puncture using a syringe attached to a 25G scalp vein needle and tubing (Exel). The right Gastrocnemius muscle (innervated by the crushed sciatic nerve) was also removed at that time. Ten animals received a sham-surgery, the procedure was identical to the sciatic nerve crush; the muscle was cut and the nerve exposed, but the nerve was not touched. The animals were sutured, monitored, and given same dose of Ibuprofen for the same number of days as the animals that received a crush to the sciatic nerve. The blood and right Gastrocnemius muscle was removed 7 days later. For different reasons, the final number of mice in each group was nine.

### Tissue handling and RNA isolation

Immediately upon removal, the Gastrocnemius muscle was flash frozen in liquid nitrogen and transferred to the −80°C freezer until processing. The muscle tissue was crushed on dry ice using a 15 mL Ultra Tissue Grinder (Fisher). Once the tissue was crushed to powder, the first buffer of the mirVana PARIS kit (Cell Disruption Buffer) was added. The samples were then sonicated using a Covaris Sonolab (Covaris Inc) with the following settings: 2 × 5% dc500 mV 100 cb.tmt for 5 s, 2 × 20% dc500 mV 50 cb.tmt for 15 s, 2 × 20% dc500 mV 100 cb.tmt for 15 s, 2 × 5% dc500 mV 100 cb.tmt for 5 s. We then continued with the protocol of the mirVana PARIS kit (Invitrogen). For muscle tissue, we measured total RNA using Nanodrop and used 1 μg in library preparation. Once blood was collected from the mice, the blood samples were spun at 2000 × *g* for 10 min (within 30 min of blood draw). Plasma was then aliquoted into 1.5 mL vials, flash frozen in liquid nitrogen, and stored at −80°C. For RNA isolation, the samples were allowed to thaw in the presence of 2 × Denaturing Buffer (mirVana PARIS kit) and then we continued with the protocol from the kit. We used the mirVana PARIS kit and followed the protocol for total RNA isolation, eluting in 100 μl water. We then precipitated the RNA in ammonium acetate (Sigma) and resuspended the RNA in 7.5 μl of RNase-free water. About 4.5 μl were then used to begin library sample preparation.

### Sequencing

Total RNA, that included small RNA, was used as the starting material in the TruSeq small RNA sample preparation from Illumina (v1.5). In order to select small RNA species from total RNA, the 3′ Illumina adaptor contains a 5′ P and in the presence of truncated T4 RNA ligase (no ATP added) it is selective for RNAs with a 3′ hydroxyl group (resulting from mature miRNA cleavage by Dicer). We used Illumina indexes 1–48, we followed the TruSeq protocol exactly and used 12 cycles of PCR amplification for gastrocnemius muscle and 15 cycles for plasma. Each library was examined on the bioanalyzer after library preparation to ensure that the samples were the proper size, had little adaptor contamination, and to estimate the sample concentration. If there was too much adaptor, the library could be rerun on a 6% TBE gel and re-purified away from the adaptor band. About 5000 pM of 10–24 samples were added to each pool, each pool was loaded at 9 pM concentration per lane for version 2 flow cells and 5 pM for version 3 flow cells. We used the bioanalyzer for calculating pM. We have noticed that the correlation between pM loaded and cluster density can vary greatly depending on the person that prepares the library. Therefore, for all of our experiments only one person prepared the libraries for the entire experiment. Using these parameters, we get an average of 415–710 clusters per mm^2^ per flow cell lane. miRNA sample libraries do not contain enough nucleotide diversity for the phasing and pre-phasing to be accurately calculated. Therefore, on all of our flow cells we dedicate one entire lane to a PhiX control, this allows the software to calculate the phasing and pre-phasing values for the whole flow cell. We processed our samples using TruSeq SBS Kit (v3) for 50 cycles of sequencing and for 7 cycles of the indexing read. The Q30 scores stayed above 90% throughout the sequencing run.

### Post-sequencing analysis pipeline

#### Sequence generation and pre-alignment filtering

Raw sequences were obtained and were de-multiplexed using the Illumina pipeline CASAVA v1.8. The FastQC^1^ and FASTX toolkit^2^ were used for Quality Check [ensured that fastq reads are in entirely normal (green tick:  ≥ Q28) range in the QC report] and to preprocess the reads prior to mapping respectively. The fastx_clipper tool was employed to remove the Illumina three prime adapter (TGGAATTCTCGGGTGCCAAGG) sequences and retaining a minimum read length of 18 bp after clipping.

#### miRNA mapping tools

We used MiRDeep2 (Friedländer et al., 2012), miRNAKey (v1.2; Ronen et al., 2010), and miRExpress (V2.1.3; Wang et al., 2009) for the analysis. All the runs were carried out using the default parameters suggested by the creators of the tools and allowing up to one single nucleotide variation (SNV).

#### miRDeep2

Clipped reads were aligned using mapper.pl to Mouse genome (mm9) and miRBase_v18 (mmu sequences) and further processed using miRDeep2.pl scripts. The csv files for miRNA expression from mirDeep2 were used for further analysis.

#### miRNAKey

Incorporates the Seq-EM algorithm to optimize the distribution of multiple aligned reads among the miRNAs expressed, and does not discard them. Output is read counts and RPM index (the Read Count normalized to a million mapped reads in the input file) values obtained by mapping against mature miRNA sequences of mouse.

#### miRExpress

Alignments of the reads are carried out against mature miRNAs from the reference genome (mouse) based on the Smith–Waterman algorithm. The read counts for each miRNA aligned were used for the downstream analysis.

### qRT-PCR

We ordered custom TaqMan MiRNA Assays from Applied Biosystems using unique miRNA sequences identified in reads sequenced in our samples by miRDeep2 that did not align with known mouse miRNA sequences in miRBase. These samples received a range of miRDeep2 scores, and were present in every sample. qRT-PCR was preformed using RNA from the right gastrocnemius muscle, 10 ng of total RNA (that includes the small RNA) was put in the reverse transcription (RT) reaction. The three unique sequences were: 5′-ucaggucccuguucgggcgcca-3′, 5′-ucacccuggacugacucucagg-3′, and 5′-agccccucugagacucugaaaga-3′. The RT reaction and PCR amplification were performed according to Taq protocol from Applied Biosystems using a Roche 480 light cycler. The RT reaction was diluted 1:15, in the Universal PCR Master Mix with no AmpErase UNG, according to the Applied Biosystems protocol. Our two positive loading controls were: snoRNA55 and snoRNA135. We also ran a no template control, in every case the no template control did not cross threshold and in every case our positive controls did call. We followed the same protocol for the qPCR validation experiments with tissue. In this case U6 was used as a control.

*C. elegans* miRNAs were used to examine sensitivity to changes in expression detectable by sequencing. *C. elegans* miRNAs *cel-miR-39, cel-miR-54, and cel-miR-238* (ordered as custom RNA oligonucleotides from IDT). A mix of these miRNAs at 25 fmol each was prepared and flash frozen in 10 μl aliquots. A volume of 1.5 μl of the mix was added to 120 μl of water. About 1.67 μl were then used in the RT reaction (5 μl reaction). About 28.9 μl of water were added to the cDNA, and 2.25 μl were used in the Taq reaction (as in Mitchell et al., 2008).

We then diluted the reaction in half and measured the *C*_p_ values for both.

### Statistical analysis

Differential expression of miRNA read counts identified by miRDeep2, miRNAKey, and miRExpress was performed using two packages designed to work with RNA based read count data. Two groups were considered for paired comparisons: (i) samples receiving sham-surgery, and (ii) samples with nerve crush.

EdgeR implementation utilizes a negative binomial distribution to model discrete count data. Although EdgeR does not transform counts to normalized RPKM values, the read count data is normalized for compositional bias in sequenced libraries and for differences between libraries in sequencing depth. The data is first scaled to library size followed by normalizing the data with weighted trimmed mean of the log expression ratios, a method known as trimmed mean of M values (TMM). We then estimate dispersion of the reads counts and perform an exact test between the groups (Robinson et al., 2010).

DESeq uses a similar approach as EdgeR while extending the model to provide a better fit for the data. The data is adjusted to a common scale by normalizing it for different library size. Secondly, the data’s (miRNA) dispersion from the mean is estimated, which provides the basis for inference. The final step is to compute differential expression and estimate *p*-values (Anders and Huber, 2010).

*p*-Values were adjusted for multiple testing with Benjamini and Hochberg (1995) approach for adjusting the false discovery rate (FDR) and adjusted *p*-values were filtered at 0.05. For biological target prediction of the differentially expressed miRNAs, we used TargetScan (Lewis et al., 2005) or DIANA-microTest 3.0 (Maragkakis et al., 2009).

## Results

We used an acute nerve injury model for these experiments. We had two groups; (1) 10 six-week-old mice underwent a surgical procedure to crush the sciatic nerve in their right leg. A bulldog clip with constant pressure was placed around the sciatic nerve for 10 s. The sciatic nerve was visibly flattened, the animals were sutured and allowed to recover in their home cages for 7 days. (2) 10 six-week-old mice received a sham-surgery, the sciatic nerve was exposed in the same way as in the animals in group 1, but the nerve was not crushed. We allowed the animals to recover for 7 days. The animals were anesthetized, blood was collected by cardiac puncture at day 7, processed for plasma, aliquoted, and flash frozen in liquid nitrogen. The animals were perfused with saline and the gastrocnemius muscle was dissected and removed, flash frozen in liquid nitrogen, and crushed to powder. A miRVana RNA Isolation Kit was used to isolate total RNA, including small RNA, from muscle and plasma samples. Illumina TruSeq Small RNA library preparation was carried out using all of the RNA isolated from plasma and 1 μg of tissue RNA. The samples were given individual barcodes, pooled and loaded onto a version 2 or version 3 single read flow cell on the Illumina HiSeq 2000, clustered, and sequenced for 50 cycles plus the indexing read. At the end of these procedures, we had nine samples in each category.

Sequencing data from the Illumina HiSeq 2000 was processed and de-multiplexed using the CASAVA pipeline to generate raw fastq reads. Quality control checks on raw sequence data were carried out using the FastQC tool. Quality filtering and other pre-alignment processing steps, adapter clipping and read collapsing, were carried out using the FASTX toolkit. Post-clipped reads were then run through three different analysis packages: miRDeep2 (Friedländer et al., 2012), miRNAKey (v1.2; Ronen et al., 2010), and miRExpress (V2.1.3; Wang et al., 2009). All the runs were carried out using the optimal parameters suggested by the developers and allowing the detection of up to a SNV.

### Description of analysis tools used for alignment and read count generation

#### miRDeep2

miRDeep2 is based on the miRNA biogenesis model, the ability to predict a miRNA’s existence by detection of the mature miRNA or any one of its precursor or stem loop sequences (Friedländer et al., 2008). It consists of three modules: the miRDeep2 module identifies known and novel miRNAs in high throughput sequencing data. The miRDeep2 core algorithm calls the RNA fold tool to predict the RNA secondary structures and evaluates the structure and signature of each potential miRNA precursor. If the structure resembles a miRNA hairpin and the reads fall in the hairpin as would be expected from Dicer processing, then the potential precursor is assigned a score that reflects the likelihood of it being a genuine miRNA. The Mapper module processes raw sequences and maps the processed reads to the reference genome. The Quantifier module sums up read counts for known miRNAs in a sequencing data set. The output of this analysis is a scored list of known and novel miRNAs with their expression levels (Friedländer et al., 2012). For the comparative analysis we examined only known miRNAs.

miRDeep2 predicts novel miRNAs based on the alignment of the putative miRNA to the genome. In the results folder, miRDeep2 displays these reads and where they map to the reference genome. The more sequences associated with any part of the pre-miRNA sequence, the higher the score. The list of novel miRNAs created by miRDeep2 is generated using an algorithm that scores the new sequence based on the predictability of the up and downstream stretches of genomic DNA (pri-miRNA sequence) to form a precursor miRNA with appropriate hairpin structure (pre-miRNA). It also uses known miRNA sequences from orthologs to increase or decrease the miRNA score. The results are reported as lists of each known miRNA detected, as well as a miRDeep2 score for both the known and predicted miRNAs (Friedländer et al., 2008).

One of the most interesting features of miRDeep2 is this output of potential novel, unreported miRNAs (discovery). We chose three unique sequences predicted by miRDeep2 to be miRNAs for further evaluation with Taq qRT-PCR. The three sequences were detected in at least 10 different gastrocnemius muscle samples. The first sequence (Sequence 1) we examined was “UCAGGUCCCUGUUCGGGCGCCA,” the miRDeep2 score for this putative miRNA was very low, 0.9 on a scale from (−)10 to 10, however the number of reads detected with this sequence was fairly high. The read counts varied from 62 to 835 across the 10 samples. The sequence is most similar to mmu-miR-5097, however, there are five bases that are different. We used custom Taq probes specifically made for these putative miRNA sequences. TaqMan has high specificity; it uses two different probes to detect the particular miRNA sequence: (1) a miRNA-specific sequence for amplification and (2) a sequence-specific probe for detection of the amplified product. Our qRT-PCR results, using a custom Taq probe for the putative miRNA above, showed an average *C*_p_ value of 19.7 across the Gastrocnemius muscle samples, there was no call for the negative control (water) using these probes (Table 1). The second sequence (Sequence 2) was: UCACCCUGGACUGACUCUCAGG, with a higher miRDeep2 score of 5.4 and a range of read counts from 15 to 529. The sequence is similar to mmu-miR-712-3p, six bases are different. The average *C*_p_ value in the muscle samples for this custom probe was 36.9 (Table 1). Our negative control did not amplify or give a *C*_p_ value. The third novel sequence (Sequence 3) was: AGCCCCUCUGAGACUCUGAAAGA. This sequence did not have a large number of read counts in any samples, range 2–85 across the 10 Gastrocnemius samples. The miRDeep2 score ranged for this sequence across the samples from 0.5 to 5.5. The score appeared to be heavily influenced by the number of reads detected in the sample (a higher miRDeep2 score with higher read counts). This sequence received both the best and worst score of the three sequences we chose to examine. The custom Taq probe showed no amplification for this putative miRNA in the qRT-PCR reactions. This could be due to several reasons, but the two easiest are either the probe was inefficient at detecting low expressing miRNAs, or the predicted miRNA is not real. We would have to use additional methods to evaluate the existence of Sequence 3. In summary, two of the three novel sequences we chose to examine could be detected by TaqMan qRT-PCR. Each of the three sequences was detected by NGS in at least 10 different gastrocnemius muscle samples.

**Table 1:** **qRT-PCR results for three potential novel miRNA sequences predicted by miRDeep2**.

Sequence	Raw mapped read counts	miRDeep2, score	TaqMan *C*_p_ value
Sequence 1 ucaggucccuguucgggcgcca	Sample 1: 759 Sample 2: 62 Sample 3: 271	0.9 0.9 0.9	19.57 18.86 19.36
Sequence 2 ucacccuggacugacucucagg	Sample 1: 373 Sample 2: 44 Sample 3: 15	5.4 5.4 5.4	36.22 36.18 37.27
Sequence 3 agccccucugagacucugaaaga	Sample 1: 49 Sample 2: 2 Sample 3: 6	5.5 0.5 2.5	– – –

*Each putative miRNA sequence appeared in at least 10 different samples and each sequence had a different miRDeep2 predicted score. The raw read counts, before normalization, from three representative Gastrocnemius muscle samples are displayed. The *C*_p_ value for each sequence in each sample are reported. The same total RNA concentration was used for each sample. The probes for Sequence 3 failed to amplify in any of the samples*.

#### miRNAKey

miRNAKey takes FASTQ sequencing files and aligns reads using BWA (Li and Durbin, 2009) to the relevant database, in our case miRBase 18. The software then can use an optional algorithm (SEQ-EM) to optimize the distribution of multiple aligned reads among the detected miRNAs, by not discarding them. It out puts both expression (alignment to reference mature miRNAs) reads counts and their normalized index based on the read count of each input sample read file. The index consists of the read count normalized to the number of (millions of) mapped reads in the input sample; RPM = (the number of reads for that sequence ÷ the total number of reads) × 1,000,000 (Ronen et al., 2010).

#### miRExpress

miRExpress consists of three modules. The first module preprocesses the sequences, e.g., adaptor removal, counting all of the sequences that are identical and collapsing them into a single sequence for alignment while retaining the information about the number of reads. The second module carries out the alignments by employing the Smith–Waterman algorithm. The third module reports miRNA expression by calculating the sum of read counts for each miRNA according to the alignment criteria chosen by the investigator. For example, the read length vs. mature miRNA length and percent identity of the alignment (Wang et al., 2009). The sequenced reads can be aligned to the mature miRNA sequence or to one of the precursor miRNA forms (pri or pre-miRNAs). Once miRExpress has performed these alignments, it takes the still unaligned reads and tries to align them to the known miRNAs present in miRBase from other species, orthologs, in an attempt to identify novel miRNAs (Wang et al., 2009).

**Figure 1:**
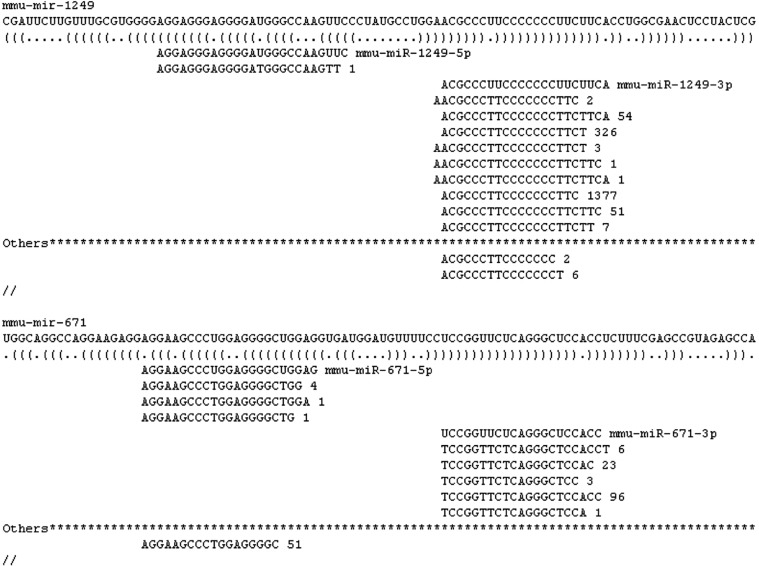
**Screen shot of miRExpress output**. Example of mature miRNA and isomiR alignment to a precursor miRNA.

An interesting and easy to use feature of the miRExpress package identifies where some of the unmapped reads go. In the results file, the miRNA reads are displayed as they align to the precursor sequence. This data reveals a category called “others” that is not counted or presented in the standard miRNA output. These are sequences that are slightly different from the mature miRNA sequence in miRBase. For instance, the 3′ end of miRNAs are often modified or shortened (Aravin and Tuschl, 2005; Landgraf et al., 2007; Westholm et al., 2011; Juvvuna et al., 2012). The sequences that match the reference miRNA but are a few nucleotides shorter or longer or mismatched by more than our one allowable SNV, are aligned and exhibited in this result folder. These slightly variable reads are called isomiRs. Figure 1 is a screen shot depicting the alignment of mmu-mir-1249-5p and 3p and mmu-mir-671-5p and 3p. The reads aligned under the mature sequence are the reads that were detected in the sample and the read counts. Under the line marked “Others” it displays sequences that were too short or too variable to be counted in the output. In the example in Figure 1, there are more miRNAs tallied in the “Others” category for mmu-miR-671-5p than align to the mature miRNA sequence. Therefore, the output to the results file is 6, rather than 51. Approximately 1–12% of the reads end up in the “others” category for Gastrocnemius muscle and are not counted as mapped reads in the output. It would be interesting to assess what the biological significance of these 3′ modifications are and whether or not they are tissue or disease specific.

### Detection of miRNAs using each application

**Figure 2:**
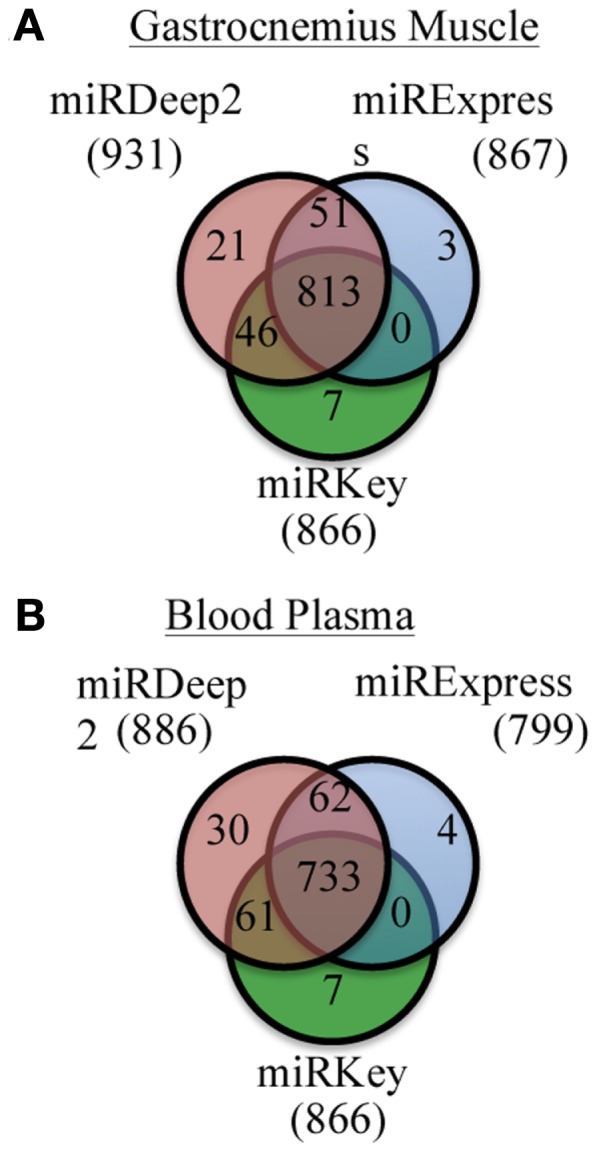
**Unique and overlapping miRNAs detected by each of the three programs for two sample types. (A)** Results of miRKey, miRDeep2, miRExpress detection of miRNAs in the Gastrocnemius muscle samples. Venn diagram displays the overlapping and unique number of miRNAs detected by each program. **(B)** Results of miRKey, miRDeep2, miRExpress detection of miRNAs in the plasma samples. Venn diagram displays the overlapping and unique number of miRNAs detected by each program.

We examined the number of miRNAs that were detected by each software tool. Figure 2 displays the number of miRNAs detected in the mouse Gastrocnemius muscle and in the plasma samples altogether by miRExpress, miRNAKey, and miRDeep2. miRDeep2 software detected and aligned more miRNAs than miRExpress or miRNAKey; miRExpress and miRNAKey performed more similarly. In this analysis we counted every miRNA, even those that had only one detected read. We also adjusted the output from each tool so that there were not duplicate counts for any mature miRNAs.

**Figure 3:**
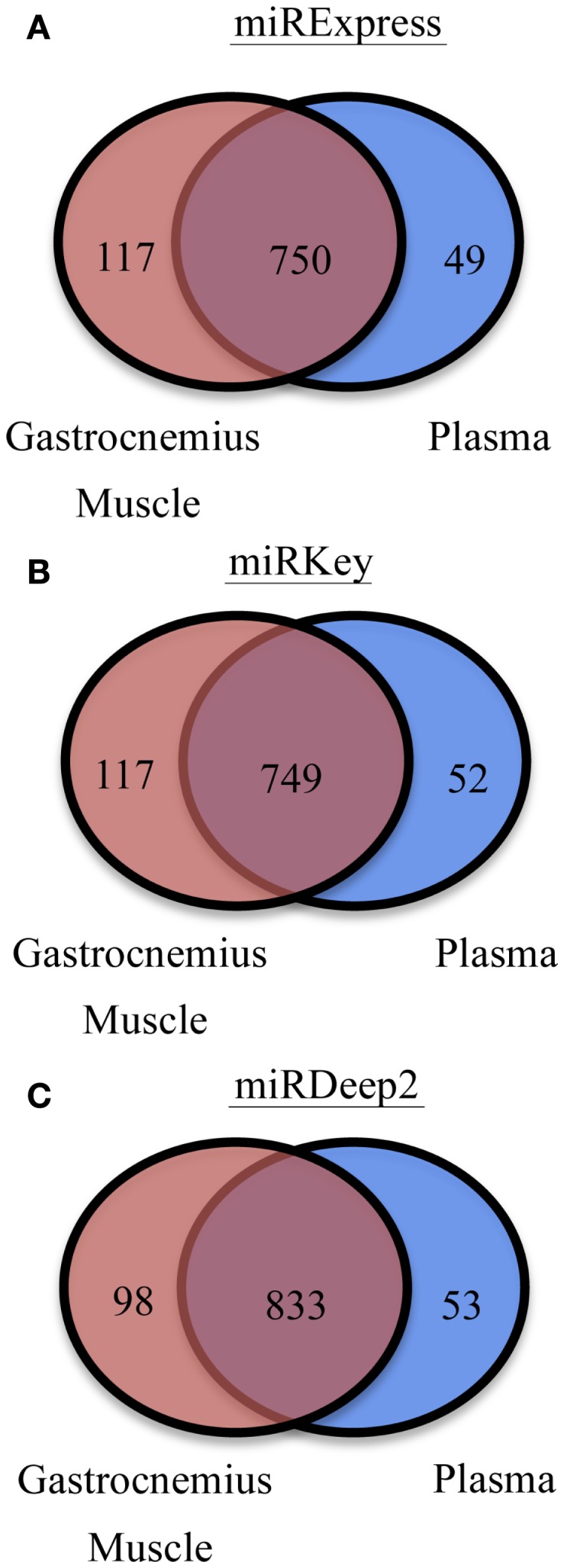
**Display of the different numbers of detectable mature miRNAs in each tissue type. (A)** miRNAs detected in Gastrocnemius Muscle and Plasma by miRExpress. **(B)** miRNAs detected in Gastrocnemius Muscle and Plasma by miRKey. **(C)** miRNAs detected in Gastrocnemius Muscle and Plasma by miRDeep2.

We were also interested in the common overlapping miRNAs and the distinct miRNAs expressed in each sample type we tested, Gastrocnemius muscle and plasma. Those results are displayed in Figure 3. There are ∼100 miRNAs expressed in the muscle that are not detected in the blood and ∼50 distinct miRNAs are identified in the plasma samples.

### Final percentage of aligned mature miRNA reads

The number of initial and mapped reads for each sample can vary greatly for small RNA sequencing libraries. Variability is due to several factors: (1) RNA isolation methods (the more small RNA present the less adaptor dimers created during library preparation), (2) different tissue sources for the RNA isolation. Blood samples perform very differently in library sample preparation than tissue samples – this is most likely due to the lesser content of small RNA present in that sample type. (3) Different amounts of adaptor dimer contamination in the library preparation that are not cut away during gel purification and are quantified and loaded onto the sequencer. (4) Difficulty in accurately measuring small pM values for each sample, 5000 pM of each sample are added to the final sample pool to be loaded on a flow cell lane, accurate measurement and loading of the final pools (9 pM) onto each lane of the sequencer. (5) Clustering efficiency is highly impacted by the size of the library. Because adaptor-only library contaminants are slightly smaller than those containing miRNAs, they will cluster more efficiently than the portion of the library containing real RNA species.

**Figure 4:**
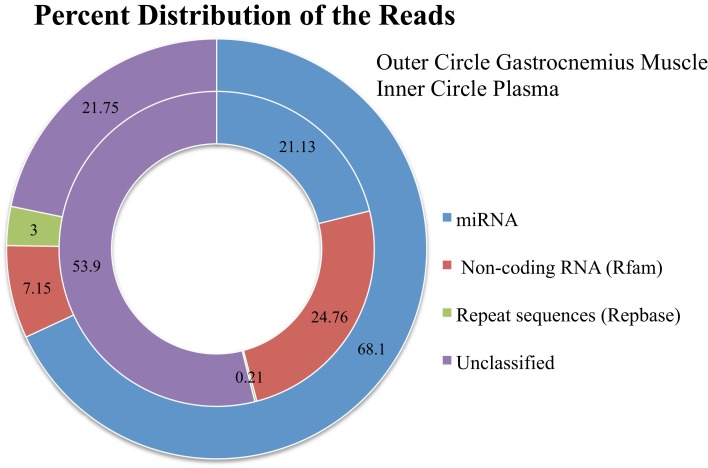
**The distribution of the total reads to different categories of known RNA sequences after removing adaptor-only and poor quality reads**.

We loaded ∼20 blood and ∼20 gastrocnemius samples onto the sequencer, ∼20 samples per lane. From our library sample preparation of the Gastrocnemius muscle, we had an average of 6,565,492 initial reads per sample (median 4,557,315; range 1,604,969–24,170,583). This wide range in the initial sequences is due to the variability listed above. There was an average of 5,634,555 post-clipped reads per sample (post-clipped median 4,007,170; range 1,306,238–18,587,936). Post-clipped reads = adaptor was trimmed, adaptor-only reads and reads that were too short were removed. The number of mapped reads that align to mature miRNA sequences from the post-clipped reads depends in part on the analysis software used: miRNAKey, miRDeep2, or miRExpress, and the tissue type (Figures 2 and 3). And in large part to the other categories of RNA sequenced in our samples (Figure 4).

Plasma sample preparation was performed identically to the Gastrocnemius muscle RNA, however, we had less initial starting material and took all of the RNA isolated from the plasma samples forward into the library sample preparation. The samples had an average of 5,948,616 initial reads (median 5,443,029; range 690,892–16,535,938). 4,852,021 post-clipped reads (median 4,767,011; range 345,433–14,894,207). The percentage of the sequences going to other categories of RNA was higher for the blood (Figure 4).

From the post-clipped reads we then align all of the sequences to miRBase. On average we get 68% mapped reads for the Gastrocnemius muscle and 21% mapped reads from the plasma samples (Figure 4). We examined what type of reads made up the rest of the post-clipped sample library. We found that Rfam (RNA family database) accounted for another 7% of the reads for the Gastrocnemius muscle and ∼25% for the plasma. RepBase (repeat sequences) made up 3% of the muscle and 0.21% of the plasma sample library. The unclassified reads (Figure 4) made up the second largest group of reads in the library and includes several potential sequence types. A portion of them may come from mature miRNAs with 3′ modifications or RNA editing of the mature miRNA sequence so that it is not counted in the other databases (∼1–12% of the total reads). Many of the reads are partial mRNA sequences. And a portion of them map to the genome, but cannot be currently classified as part of the other categories.

### The effect of depth of coverage on miRNA detection

While sequencing costs have gone down, the cost of sequencing is still not trivial. The depth of sequencing coverage is clearly linked to a better estimation of the expressed miRNAs and downstream differential expression analysis. High sequencing depth should enrich the number of detected miRNAs and lower the false positive rate for significant differentially expressed miRNAs. However, what is a good depth of coverage for small RNA sequencing? It can become very costly to sequence small RNA samples over and over to get the level of coverage desired. As shown in Figure 4, depending on your library preparation and the sample type, only 21–68% of our reads mapped to mature miRNAs in miRBase. Similar percentages of mapped reads have been observed by other researchers (Eipper-Mains et al., 2012). We wanted to try and understand how depth of coverage would change our detection of miRNAs. For each sample type, muscle, and plasma, there is a significant amount of variability in the number of initial reads and reads that map to mature miRNAs. Therefore, we examined the effects of increasing mapped read counts on the ability to detect miRNAs in both muscle and blood. We used one sample from each tissue type with a large number of initial and mapped reads as a representative example.

**Figure 5:**
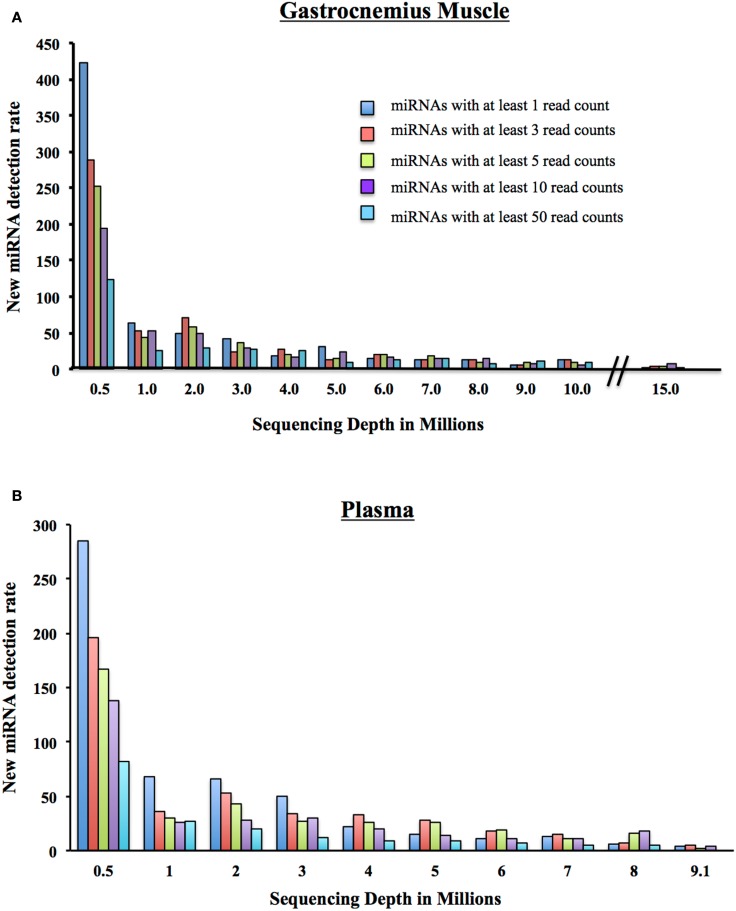
**New miRNA detection rate. (A)** As a million reads at a time are added to the sequencing depth of the Gastrocnemius Muscle, the number of newly detectable miRNAs is reduced. Displayed are the number of newly detected miRNAs with at least 1, 3, 5, 10, or 50 reads. **(B)** As a million reads at a time are added to the sequencing depth of the Plasma, the number of newly detectable miRNAs is reduced. Displayed are the number of newly detected miRNAs with at least 1, 3, 5, 10, or 50 reads.

We began with 500,000 randomly chosen post-clipped reads and mapped them to miRBase using miRDeep2. We display the number of detected miRNAs and their corresponding coverage: 1 mapped read only, at least 3 mapped reads, at least 5 mapped reads, 10 mapped reads, and 50 mapped reads. We increased the number of post-clipped reads going into the alignment incrementally and calculated the number of new miRNAs that were detected with each addition of reads (Figure 5). Not surprisingly, we found that the more we increased the input of reads, the more miRNAs we slowly included. The number of additional miRNAs detected vs. the increased number of reads required has to be weighed for each experiment. At the start of any experiment, we recommend sequencing representative control samples for each tissue type to a significant depth to examine the number of miRNAs that can be detected and determine what number of detectable miRNAs is suitable for your experiments.

### Examination of how well low depth of coverage can represent the sample distribution

**Figure 6:**
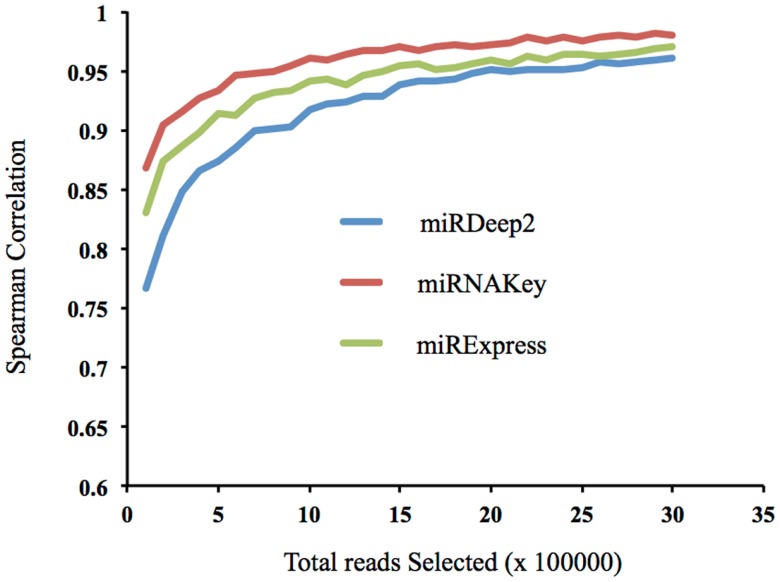
**The correlation of a subset of reads, beginning with 100,000 with the full number of mapped reads, 3.5 million**.

In addition to examining how the incremental increase in coverage altered the detection of new miRNAs in the sample, we wanted to examine how well low numbers of mapped reads represented the sample when compared with a very large number of mapped reads. In other words, how well do a small subset of the reads represent the distribution of miRNAs in the sample. This analysis uses mapped reads whereas the previous analysis used post-clipped reads. Using a plasma sample, we began with 100,000 randomly chosen mapped reads, and incrementally increased the number of reads we included by 100,000 up to 3.5 million. We then calculated how well the subset of the lower numbers of read inputs, such as 100,000, correlated with the final total of 3.5 million mapped reads (Figure 6). If the correlation is high with a low number of reads, it suggests that low coverage may be sufficient to represent the sample. Selecting reads randomly (instead of miRNAs) preserves the original distribution of reads from the sequencer. We report the Spearman correlations using the three different tools: miRDeep2, miRNAKey, and miRExpress. The correlation becomes fairly stable at ∼1.5 million random reads, correlation coefficient of 0.97 for miRNAKey. Our main observation was that increasing the reads from 1 to 3 million reads only increased the Spearman correlation by 0.05.

### Analysis of the nerve crush data

Our final analysis consists of data from the mice that received a sciatic nerve crush compared with mice that received a sham-surgery. The intent of this experiment was to identify miRNAs specific to nerve crush/injury. We used only the reads that mapped to known miRNAs for the analysis. We used each of the three software packages: miRNAKey, miRDeep2, and miRExpress, and analyzed the data using both DESeq and EdgeR (Tables 2–5).

**Table 2:** **Differentially expressed genes in the Gastrocnemius muscle detected using EdgeR for sham-surgery vs. nerve crush**.

mirDeep2			miRExpress			miRNAKey		
mirName	FC (log2)	*p-*Value	mirName	FC (log2)	*p-*Value	mirName	FC (log2)	*p-*Value
mmu-mir-3969	4.471	0.000	mmu-miR-31-5p	2.187	0.010	mmu-miR-5115	−2.552	0.001
mmu-miR-31-5p	2.406	0.000	mmu-miR-483-5p	−2.496	0.010	mmu-miR-31-5p	2.430	0.001
mmu-mir-5115	−2.259	0.001	mmu-miR-1249-3p	−2.098	0.011	mmu-miR-5105	−2.862	0.008
mmu-mir-3962	2.696	0.003	mmu-miR-3102-3p	−2.027	0.014	mmu-miR-1249-3p	−2.059	0.016
mmu-miR-1249-3p	−2.037	0.006	mmu-miR-423-5p	−2.014	0.014	mmu-miR-1247-5p	−2.098	0.026
mmu-miR-5105	−2.084	0.010	mmu-mir-5109	−1.954	0.016	mmu-miR-3102-3p	−1.968	0.028
mmu-miR-423-5p	−1.938	0.011	mmu-miR-1298-5p	−3.896	0.028	mmu-miR-423-5p	−1.917	0.000
mmu-miR-3102-3p	−1.924	0.012	mmu-miR-1247-5p	−1.811	0.043	mmu-miR-1298-5p	−3.888	0.032
mmu-miR-1247-5p	−1.910	0.014				mmu-miR-744-5p	−1.841	0.032
mmu-miR-744-5p	−1.705	0.042						

*Values in yellow highlight indicate differentially expressed miRNAs that were identified by all three alignment tools. Blue highlight are differentially expressed miRNAs identified in two out of three tools. Results were filtered at corrected *p*-value < 0.05. The results were truncated at the top 10 differentially expressed miRNAs for miRDeep2*.

**Table 3:** **Differentially expressed miRNAs in the Gastrocnemius muscle detected by DESeq for Sham-Surgery vs. nerve crush**.

miRDeep2			miRExpress			miRNAKey		
miRNA Name	FC (log2)	*p*-Value	miRNA Name	FC (log2)	*p*-Value	miRNA Name	FC (log2)	*p*-Value
mmu-miR-3969	−4.2262	0.0098						

*Results were filtered at corrected *p*-value < 0.05*.

**Table 4:** **Differentially expressed genes in plasma detected using EdgeR for Sham-Surgery vs. nerve crush**.

mirDeep2			miRExpress			miRNAKey		
mirName	FC (log2)	*T*, *p-*Value	mirName	FC (log2)	*p-*Value	mirName	FC (log2)	*p-*Value
						mmu-miR-1a-3p	2.762	0.002
						mmu-miR-411-5p	2.516	0.006
						mmu-miR-378-3p	2.454	0.006
						mmu-miR-133a-3p	2.330	0.011
						mmu-miR-196a-5p	2.648	0.011
						mmu-miR-9-5p	2.129	0.028
						mmu-miR-381-3p	2.190	0.028
						mmu-miR-153-3p	2.426	0.028
						mmu-miR-708-5p	2.624	0.030
						mmu-miR-411-3p	2.641	0.038

*Results were filtered at corrected *p*-value < 0.05*.

**Table 5:** **Differentially expressed miRNAs in plasma detected by DESeq for Sham-Surgery vs. nerve crush**.

miRDeep2			miRExpress			miRNAKey		
miRNA Name	FC (log2)	*p*-Value	miRNA Name	FC (log2)	*p*-Value	miRNA Name	FC (log2)	*p*-Value
						mmu-miR-133a-3p	−2.49	0.028
						mmu-miR-1a-3p	−3.01	0.028
						mmu-miR-378-3p	−2.65	0.028

*Results were filtered at corrected *p*-value < 0.05*.

In the Gastrocnemius muscle data, there was a lot of overlap (yellow and blue highlighted miRNAs) between the three tools using EdgeR for analysis. With DESeq, we only got one significantly different miRNA. The analysis of the plasma found significantly different miRNAs using miRNAKey only. The plasma samples may have been more variable since they had overall fewer mapped reads per sample. It is also possible that a nerve crush injury, 7 days after the insult is less detectable in blood. It would be interesting to examine an earlier time point after nerve injury to see if more miRNAs were detectable in blood. Perhaps changes in miRNA expression, in response to nerve crush, remain local in the tissue and do not become elevated in the blood. We would like to explore this possibility further because researchers have begun looking for miRNA biomarkers to indicate nerve-related injury and disease.

Because we were interested in the altered expression of miRNAs in response to nerve injury, and what the underlying relevance might be to injury and repair processes, we examined the predicted gene targets. We used miRNA Targets and Expression to examine the predicted targets for the five differentially expressed miRNAs that appeared across all 3 analysis tools using EdgeR (mmu-mir-31-5p, mmu-mir1249-3p, mmu-mir-423-5p, mmu-mir-3102-3p, mmu-mir-1247-5p; Enright et al., 2003; John et al., 2005; Betel et al., 2008, 2010).

### Validation

We next wanted to validate the sequencing results using TaqMan qRT-PCR. However, the use of qPCR to validate sequencing results in these, and other, experiments has not proven to be straight forward. There are several reasons for this: (1) sensitivity, we have found that qPCR is not as sensitive as sequencing. qPCR has a large dynamic range, measuring a few to millions of molecules. But because the scale is logarithmic. The measurement between a few molecules and millions fits on a scale between 5–30 cycles. We display in Table 6 below, that although there is no detectable difference in *C*_p_ values for qPCR, there are differences that can be identified by sequencing, (2) cross-reactivity, the probes may create some variability when comparing to sequencing due to closely related isomiRs or miRNAs with single nucleotide changes that qPCR cannot differentiate, (3) qPCR and sequencing are very different platforms with different biases (loading, reference gene, ligation, probe design, primer melting temperatures), it is difficult to compare the results directly, (4) miRNAs are very small and the sequence used for the RT reaction is limited by the sequence of the miRNA. Therefore, the probe may not have optimal melting temperatures or ideal linear amplification qualities. Nevertheless, we report the average delta *C*_p_ values in the table below, using a *t* test the delta *C*_p_ values are not significantly different. While the qPCR results are not significantly different between groups, we also display the read counts detected from sequencing the two groups. Using a *t* test, the read counts are significant at *p* < 0.03.

**Table 6:** **qPCR results**.

**GASTROCNEMIUS MUSCLE – AVERAGE DELTA *C*_p_ VALUES**									
	**mmu-31**		**mmu-1247-5p**				**mmu-3102-3p**		**mmu-3969**
Crush	8.6		9.4				7.5		7.8
Sham	7.8		8.8				8.2		9.4
**MMU-31 DELTA *C*_p_ VALUES FOR 5 MUSCLE SAMPLES**									
Crush	8.50		11.40			7.74	8.91		8.65
Sham	7.32		9.47			10.17	6.36		5.89
**SEQUENCING READ COUNTS FOR MMU-31**									
Crush	13	4	125	47	55	8	20	31	15
Sham	47	525	55	15	11	274	257	229	
**PLASMA – AVERAGE DELTA *C*_p_**									
		**mmu-133-3p**					**mmu-1a-3p**		
Crush		0.74					1.68		
Sham		2.99					0.03		

*The first table is the average *C*_p_ values for the crush and sham groups for top differentially expressed miRNAs. The first three are shared across all groups using EdgeR mmu-31, 1247, 3102. mmu-3969 was the only miRNA detected by DESeq for the muscle samples. Below that we show the delta *C*_p_ values for five samples from each group. Then we display the sequencing read counts for each muscle for mmu-31. Forth, we show the average delta *C*_p_ for the plasma miRNAs. The reference was U6*.

In order to further illustrate some of the problems associated with qPCR validation, we used three *C. elegans* synthetic miRNAs: cel-39, cel-54, cel-238. We put 1.5 μl of a 25 fmol stock of each one in a mix and put them in 120 μl of water. We carried 1.67 μl forward into three separate and specific RT reactions. We then used a specific Taq probe for each. We had the following *C*_p_ values: cel-39 (*C*_p_ 17.9), cel-238 (20.3), cel-54 (18.3). We diluted the RT reaction in half and got the following *C*_p_ values: cel-39 (17.3), cel-238 (21.8), cel-54 (18.7). Taq is not sensitive enough to pick up the difference in half the molecules at this range.

## Discussion

We began our sequencing studies with the intent to identify specific miRNAs related to nerve injury. Our goal was to examine miRNAs that were expressed at significantly different levels between mice with a traumatic injury, inflammation, and damage associated with an injury where the skin and muscle (quadriceps) were cut and then repaired (sham-surgery) compared with miRNAs differentially expressed in mice that received the same insult plus a crushed nerve (sciatic). The experiment was to detect differentially expressed miRNAs resulting specifically from nerve injury in an affected muscle downstream of the crushed nerve. The Gastrocnemius muscle is innervated by the sciatic nerve, but was not directly damaged by our surgical procedures. We isolated RNA from the Gastrocnemius muscle and peripheral blood plasma and used NGS as our assay.

We tested the ability of miRDeep2 to predict novel miRNAs by attempting to validate the miRNA sequence with a second type of detection, custom TaqMan qRT-PCR probes. We found that the appearance of the novel miRNA sequence in multiple samples, with a high number of detected reads, helped lend confidence to the existence of the miRNA. We were unable to detect one of the miRNAs in any of the samples, either the probes were inefficient at detecting that sequence at that level of expression, or the sequence is not a true miRNA. Additional tests are required.

There was a significant amount of upfront information that needed to be collected before we could carry out our experiments. We needed to know how each sample type (tissue vs. plasma) would perform on the sequencer. For instance, what percent of the initial sequenced reads would map to known mature miRNAs? How does the sequence coverage affect miRNA detection, and how many mapped reads are required to best represent the sample? We also wanted to explore some of the most cited software tools for aligning and reporting mature miRNA counts. We also examined some of the additional features provided by each software package. All of these pieces of information, weighed against the resources and costs required to sequence the samples to a suitable depth, determined how the sequencer was loaded; the number of barcoded samples per lane and how the data was analyzed.

One of the most surprising outcomes from our sequencing data was the overall low percentage of mapped reads, on average ∼21–68% of all of the reads mapped to known mature miRNAs. There were several other categories of RNA that took up a large proportion of our reads. This made it more difficult to achieve a high depth of coverage, especially for plasma samples. A large portion of the reads went to an unassigned category. These could be mRNA, or they aligned to the genome but were unknown, or contamination.

We went on to consider the number of new miRNAs detected with the addition of a million reads. Many researchers use a minimum of 10 read counts for a particular miRNA as a cutoff for inclusion in the final analysis (Dhahbi et al., 2011; Hu et al., 2011). Therefore, if we look only at the addition of new miRNAs that have at least 10 reads (Figure 5A), at a total of 5 million reads, you include 24 new miRNAs. If you add a million post-clipped reads making the total now 6 million reads, you include an additional ∼17 new miRNAs with at least 10 reads are included. At 7 million, 14 new miRNAs, at 8 million (14 miRNAs), 9 million (8 miRNAs), and at 10 million reads (6 miRNAs). That is a total of 83 new miRNAs detected between 5 and 10 million reads for the Gastrocnemius muscle. For the plasma sample, we add 58 newly detected miRNAs between 5 and 9 million reads. We conclude, therefore, that individual investigators need to examine the properties of their samples to decide what depth of coverage would be best for their experiments.

When we looked at the Spearman Correlation and how well 1.5 million mapped reads correlated with more than double that number (3.5 million mapped reads), it was 0.97. If this were a typical plasma sample with ∼20% of the initial reads mapping to known miRNAs, 3.5 million mapped read counts would come from ∼17.5 million initial read counts. To get just the 1.5 million mapped reads, the input reads would have to be 7.5 million reads from the sequencer. The numbers of reads required to get just a million mapped reads quickly becomes very high and difficult to support.

Taking all of these things into consideration along with the cost and resources necessary to continue sequencing each of these samples, the samples included in the Gastrocnemius muscle analysis all had at least 1,000,000 mapped reads, except two samples that each had >650,000 mapped reads. We did have to settle for a much smaller number of mapped reads in the plasma samples. The average number of reads that went into the analysis at the end was ∼500,000 reads for each sample.

Once we had our samples for the analysis, we examined the outputs of both EdgeR and DESeq, common analysis tools for sequencing data. In general, DESeq identified many fewer significant differentially expressed miRNAs. Possibly because of the lower read count increasing the variability in the plasma samples, or possibly due to biology, and only a very small affect of nerve injury in the leg on miRNA changes in the blood, but there were many fewer differentially expressed miRNAs detected in plasma.

We attempted to validate our sequencing findings using qRT-PCR. In many ways qPCR is inferior to sequencing when measuring small, but significant, changes in RNASeq or in miRNASeq. qPCR has been an excellent method for validating data such as microarrays, but perhaps we need to identify a new way to validate sequencing results. We illustrated several reasons for this above. Among the reasons we mentioned, the logarithmic nature of qPCR results make it difficult to detect modest changes in molecule numbers. In our example, cutting the reaction in half made no detectable difference to the qPCR *C*_p_ values in the ranges we detect miRNAs.

There is a lot of value in using NGS technologies to assess miRNA profiles. Investigators can detect, isomiRs, subtle changes in miRNA expression and potential novel undiscovered miRNAs. There are many tools that can be used to analyze the data. We investigated some of them using a dataset for nerve injury.

## Conflict of Interest Statement

The authors declare that the research was conducted in the absence of any commercial or financial relationships that could be construed as a potential conflict of interest.

## Footnotes

^1^http://www.bioinformatics.babraham.ac.uk/projects/fastqc

^2^http://hannonlab.cshl.edu/fastx_toolkit

## References

[B1] Anders S. Huber W. (2010). Differential expression analysis for sequence count data. Genome Biol. 11, R106 10.1186/gb-2010-11-10-r106 20979621 PMC3218662

[B2] Aravin A. Tuschl T. (2005). Identification and characterization of small RNAs involved in RNA silencing. FEBS Lett. 579, 5830–5840 10.1016/j.febslet.2005.08.009 16153643

[B3] Benjamini Y. Hochberg Y. (1995). Controlling the false discovery rate: a practical and powerful approach to multiple testing. J. R. Statist. Soc. 57, 289–300

[B4] Betel D. Koppel A. Aglus P. Sander C. Leslie C. (2010). Comprehensive modeling of microRNA targets predicts functional non-conserved and non-canonical sites. Genome Biol. 11, R90 10.1186/gb-2010-11-8-r90 20799968 PMC2945792

[B5] Betel D. Wilson M. Gabow A. Marks D. S. Sander C. (2008). The microRNA org resource: targets and expression. Nucleic Acids Res. 36, D149–D153 10.1093/nar/gkn293 18158296 PMC2238905

[B6] Dhahbi J. M. Atamna H. Boffelli D. Magis W. Spindler S. R. Martin D. I. (2011). Deep sequencing reveals novel microRNAs and regulation of microRNA expression during cell senescence. PLoS ONE 6:e20509 10.1371/journal.pone.0020509 21637828 PMC3102725

[B7] Ebert M. S. Sharp P. A. (2012). Roles for microRNAs in conferring robustness to biological processes. Cell 149, 515–524 10.1016/j.cell.2012.04.005 22541426 PMC3351105

[B8] Eipper-Mains J. E. Eipper B. A. Mains R. (2012). Global approaches to the role of miRNAs in drug-induced changes in gene expression. Front. Genet. 3:109 10.3389/fgene.2012.00109 22707957 PMC3374462

[B9] Enright A. J. John B. Gaul U. Tuschi T. Sander C. Marks D. S. (2003). MicroRNA targets in drosophila. Genome Biol. 5, R1 10.1186/gb-2003-5-1-r1 14709173 PMC395733

[B10] Espinoza-Lewis R. A. Wang D. Z. (2012). MicroRNAs in heart development. Curr. Top. Dev. Biol. 100, 279–317 10.1016/B978-0-12-387786-4.00009-9 22449848 PMC4888772

[B11] Friedländer M. R. Chen W. Adamidi C. Maaskola J. Einspanier R. Knespel S. (2008). Discovering microRNAs from deep sequencing data using miRDeep. Nat. Biotechnol. 26, 407–415 10.1038/nbt1394 18392026

[B12] Friedländer M. R. Mackowiak S. D. Li N. Chen W. Rajewsky N. (2012). miRDeep2 accurately identifies known and hundreds of novel microRNA genes in seven animal clades. Nucleic Acids Res. 40, 37–52 10.1093/nar/gkr1251 21911355 PMC3245920

[B13] Hu H. Y. Guo S. Xi J. Yan Z. Fu N. Zhang X. (2011). MicroRNA expression and regulation in human, chimpanzee, and macaque brains. PLoS Genet. 7:e1002327 10.1371/journal.pgen.1002327 22022286 PMC3192836

[B14] John B. Enright A. J. Aravin A. Tuschi T. Sander C. Marks D. S. (2005). Human microRNA targets. PLoS Biol. 3:e264 10.1371/journal.pbio.0030264 PMC521178 15502875

[B15] Juvvuna P. K. Khandelia P. Lee L. M. Makeyev E. V. (2012). Argonaute identity defines the length of mature mammalian microRNAs. Nucleic Acids Res. 40, 6808–6820 10.1093/nar/gks293 22505576 PMC3413106

[B16] Landgraf P. Rusu M. Sheridan R. Sewer A. Iovino N. Aravin A. (2007). A mammalian microRNA expression atlas based on small RNA library sequencing. Cell 129, 1401–1414 10.1016/j.cell.2007.04.040 17604727 PMC2681231

[B17] Lewis B. P. Burge C. B. Bartel D. P. (2005). Conserved seed pairing, often flanked by adenosines, indicates that thousands of genes are microRNA targets. Cell 120, 15–20 10.1016/j.cell.2004.12.035 15652477

[B18] Li H. Durbin R. (2009). Fast and accurate short read alignment with Burrows-Wheeler transform. Bioinformatics 25, 1754–1760 10.1093/bioinformatics/btp100 19451168 PMC2705234

[B19] Luís A. L. Amadoc S. Geunad S. Rodrigues J. M. Simões M. J. Santos J. D. (2007). Long-term functional and morphological assessment of a standardized rat sciatic nerve crush injury with a non-serrated clamp. J. Neurosci. Methods 163, 92–104 10.1016/j.jneumeth.2007.02.017 17397932

[B20] Maragkakis M. Reczko M. Simossis V. A. Alexiou P. Papadopoulos G. L. Dalamagas T. (2009). DIANA-microT web server: elucidating microRNA functions through target prediction. Nucleic Acids Res. 37, W273–W276 10.1093/nar/gkp292 19406924 PMC2703977

[B21] Mazzer P. Y. Barbieria C. H. Mazzer N. Fazan V. P. (2008). Morphologic and morphometric evaluation of experimental acute crush injuries of the sciatic nerve of rats. J. Neurosci. Methods 173, 249–258 10.1016/j.jneumeth.2008.06.019 18644327

[B22] Mitchell P. S. Parkin R. K. Kroh E. M. Fritz B. R. Wyman S. K. Pogosova-Agadjanyan E. L. (2008). Circulating microRNAs as stable blood-based markers for cancer detection. Proc. Natl. Acad. Sci. U.S.A. 105, 10513–10518 10.1073/pnas.0804549105 18663219 PMC2492472

[B23] Natarajan R. Putta S. Kato M. (2012). MicroRNAs and diabetic complications. J. Cardiovasc. Transl. Res. 5, 413–422 10.1007/s12265-012-9368-5 22552970 PMC3396726

[B24] Nikitina E. G. Urazova L. N. Stegny V. N. (2012). MicroRNAs and human cancer. Exp. Oncol. 34, 2–8 22453141

[B25] Osanto S. Qin Y. Buermans H. P. Berkers J. Lerut E. Goeman J. J. (2012). Genome-wide MicroRNA expression analysis of clear cell renal cell carcinoma by next generation deep sequencing. PLoS ONE 7:e38298 10.1371/journal.pone.0038298 22745662 PMC3380046

[B26] Ponomarev E. D. Veremeyko T. Weiner H. L. (2012). MicroRNAs are universal regulators of differentiation, activation, and polarization of microglia and macrophages in normal and diseased CNS. Glia 61, 91–103 10.1002/glia.22363 22653784 PMC3434289

[B27] Pritchard C. C. Cheng H. H. Tewari M. (2012). MicroRNA profiling: approaches and considerations. Nat. Rev. Genet. 13, 358–369 10.1038/ni.2279 22510765 PMC4517822

[B28] Provost P. (2010). Interpretation and applicability of microRNA data to the context of Alzheimer’s and age-related diseases. Aging 2, 166–169 20375468 10.18632/aging.100131 PMC2871245

[B29] Robinson M. D. McCarthy D. J. Smyth G. K. (2010). edgeR: a Bioconductor package for differential expression analysis of digital gene expression data. Bioinformatics 26, 139–140 10.1093/bioinformatics/btp616 19910308 PMC2796818

[B30] Ronen R. Gan I. Modai S. Sukacheov A. Dror G. Halperin E. (2010). miRNAkey: a software for microRNA deep sequencing analysis. Bioinformatics 26, 2615–2616 10.1093/bioinformatics/btq493 20801911

[B31] Schroen B. Heymans S. (2012). Small but smart – microRNAs in the centre of inflammatory processes during cardiovascular diseases, the metabolic syndrome, and ageing. Cardiovasc. Res. 93, 605–613 10.1093/cvr/cvr268 21994347

[B32] Semenov D. V. Baryakin D. N. Brenner E. V. Kurilshikov A. M. Vasiliev G. V. Bryzgalov L. A. (2012). Unbiased approach to profile the variety of small non-coding RNA of human blood plasma with massively parallel sequencing technology. Expert Opin. Biol. Ther. S1, S43–S51 10.1517/14712598.2012.679653 22509727

[B33] Shantikumar S. Caporali A. Emanueli C. (2012). Role of microRNAs in diabetes and its cardiovascular complications. Cardiovasc. Res. 93, 583–593 10.1093/cvr/cvr300 22065734 PMC3291087

[B34] Van Nieuwerburgh F. Soetaert S. Podshivalova K. Wang E. A. Schaffer L. Deforce D. (2011). Quantitative bias in illumina TruSeq and a novel post amplification barcoding strategy for multiplexed DNA and small RNA deep sequencing. PLoS ONE 6:e26969 10.1371/journal.pone.0026969 22046424 PMC3203936

[B35] Wang F. Li L. Liu L. Li H. Zhang Y. Yao Y. (2012). High-throughput sequencing discovery of conserved and novel microRNAs in Chinese cabbage (*Brassica rapa* L. ssp. pekinensis). Mol. Genet. Genomics 287, 555–563 10.1007/s00438-012-0699-3 22643909

[B36] Wang W. C. Lin F. M. Chang W. C. Lin K. Y. Huang H. D. Lin N. S. (2009). miRExpress: analyzing high-throughput sequencing data for profiling microRNA expression. BMC Bioinformatics 10:328 10.1186/1471-2105-10-328 19821977 PMC2767369

[B37] Weiland M. Gao X. H. Zhou L. Mi Q. S. (2012). Small RNAs have a large impact: circulating microRNAs as biomarkers for human diseases. RNA Biol. 9, 850–859 10.4161/rna.20378 22699556

[B38] Westholm J. O. Ladewig E. Okamura K. Robine N. Lai E. C. (2011). Common and distinct patterns of terminal modifications to mirtrons and canonical microRNAs. RNA 18, 177–192 10.1261/rna.030627.111 22190743 PMC3264906

